# Genomic Anomaly Detection with Functional Data Analysis

**DOI:** 10.3390/genes16060710

**Published:** 2025-06-15

**Authors:** Ria Kanjilal, Andre Luiz Campelo dos Santos, Sandipan Paul Arnab, Michael DeGiorgio, Raquel Assis

**Affiliations:** 1 Department of Electrical Engineering and Computer Science, Florida Atlantic University, Boca Raton, FL 33431, USA; rkanjila@calpoly.edu (R.K.); acampelodossanto@fau.edu (A.L.C.d.S.); sarnab2020@fau.edu (S.P.A.); 2 Institute for Human Health and Disease Intervention, Florida Atlantic University, Boca Raton, FL 33431, USA

**Keywords:** anomaly detection, feature extraction, functional data analysis, isolation forest, support vector machine

## Abstract

**Background:** Genetic variation provides a foundation for understanding evolution. With the rise of artificial intelligence, machine learning has emerged as a powerful tool for identifying genomic footprints of evolutionary processes through simulation-based predictive modeling. However, existing approaches require prior knowledge of the factors shaping genetic variation, whereas uncovering anomalous genomic regions regardless of their causes remains an equally important and complementary endeavor. **Methods:** To address this problem, we introduce ANDES (ANomaly DEtection using Summary statistics), a suite of algorithms that apply statistical techniques to extract features for unsupervised anomaly detection. A key innovation of ANDES is its ability to account for autocovariation due to linkage disequilibrium by fitting curves to contiguous windows and computing their first and second derivatives, thereby capturing the “velocity” and “acceleration” of genetic variation. These features are then used to train models that flag biologically significant or artifactual regions. **Results:** Application to human genomic data demonstrates that ANDES successfully detects anomalous regions that colocalize with genes under positive or balancing selection. Moreover, these analyses reveal a non-uniform distribution of anomalies, which are enriched in specific autosomes, intergenic regions, introns, and regions with low GC content, repetitive sequences, and poor mappability. **Conclusions:** ANDES thus offers a novel, model-agnostic framework for uncovering anomalous genomic regions in both model and non-model organisms.

## 1. Introduction

Observed genetic variation arises from a combination of biological and technical factors [1]. Biological sources include mutation, recombination, gene flow, genetic drift, and natural selection. Mutation generates novel genetic variants that can be shuffled into new haplotypes by recombination, spread across populations by gene flow, lost at random by genetic drift, or purged or retained by natural selection [2,3]. Detecting genomic regions affected by these and other evolutionary processes can therefore provide insights into both evolutionary history and disease mechanisms [1,3,4]. In contrast, technical sources of genetic variation stem from errors introduced during DNA isolation and sequencing [5,6], mapping [7], and contamination [8]. These artifacts can distort measurements and bias downstream analyses, making their identification essential for accurately interpreting patterns of genetic variation.

Genetic variation is often quantified using summary statistics, which are concise measures that capture diversity within and among populations [1,3,9,10,11,12,13,14,15,16]. Over the past several decades, these statistics have been used to identify numerous genomic regions of interest, including those impacted by natural selection [12,13,17]. However, because they do not explicitly incorporate haplotype structure and often assume independence among loci [13,18], classical summary statistics [9,10] may not fully represent the effects of linkage disequilibrium [19]. To account for linkage disequilibrium, researchers have developed advanced statistics that utilize haplotype information or patterns of genetic variation across the genome to address autocovariation in diversity [20,21]. For example, likelihood-based methods offer advantages over classical summary statistics by leveraging the spatial distribution and marginal allele frequencies of linked sites [22,23,24,25,26]. Yet, even these approaches often rely on a limited set of user-selected features or statistics, which may constrain their ability to detect more complex or unanticipated patterns.

In contrast, machine learning algorithms can operate with or without summary statistics, providing a unique edge over likelihood-based approaches. They are also well-suited for analyzing high-dimensional genomic data, automatically learning relevant features, capturing complex relationships, detecting subtle patterns in data, and enhancing the flexibility and adaptability of analyses [27]. A key strength of machine learning lies in its capacity to establish interpretable links between input and output variables, achieved through the learned weights of an optimal model architecture [28,29]. Moreover, machine learning prioritizes predictive accuracy over explicit parameter estimation, allowing it to uncover meaningful patterns even when the underlying models are incomplete or uncertain [27,30].

Many powerful machine learning methods have been developed to identify genomic footprints of specific evolutionary processes [27,29,31,32,33,34,35,36,37,38,39,40,41,42,43,44,45,46,47,48,49]. However, it is equally important to locate anomalous genomic regions irrespective of the forces driving them, as this may broaden the range of detectable biological phenomena and help flag problematic regions of the genome. This task falls under the domain of anomaly detection, which aims to identify data points or outliers that deviate from expectations defined by the distribution of most observed data [50]. Several strategies for anomaly detection have been utilized in evolutionary genomics, such as clustering, dimensionality reduction, and distance-based methods [51,52,53,54]. The overarching goal of such methods is to identify unusual patterns in a set of measured features extracted from raw genomic data. Because these features capture essential information, their quality plays a critical role in determining model performance and predictive accuracy [55].

In this context, the statistical framework of functional data analysis (FDA) has garnered attention in evolutionary genomics for its ability to extract meaningful features and patterns from continuous data [56]. FDA treats measured values as the outputs of underlying functions, thereby capturing the inherent relatedness among data points [57,58]. Because it enables the analysis of complex variation over time or space, FDA can detect temporal and spatial autocovariance in genetic data that may be overlooked by traditional summary statistics [39,43]. Methods that directly model feature autocovariation have been successfully integrated into modern machine and deep learning frameworks, achieving notable performance in fault and anomaly detection applications [59,60,61]. Therefore, by leveraging the power of FDA for anomaly detection, researchers can uncover genomic regions influenced by a diverse range of evolutionary processes.

In this study, we used statistical techniques—specifically moments and FDA–to extract features from consecutive genomic windows of summary statistics computed from single nucleotide polymorphisms (SNPs). We introduce ANDES (ANomaly DEtection using Summary statistics), a suite of methods that merge the power of unsupervised anomaly detection algorithms with feature extraction techniques that model genomic autocovariation. This framework identifies aberrant genomic regions potentially shaped by biological or technical factors without requiring knowledge of the underlying genetic and demographic factors shaping variation in a given study system. Specifically, ANDES assigns anomaly scores in the form of *p*-values to individual genomic positions and then applies a significance threshold to flag regions as anomalous. In an empirical analysis of whole-genome sequences from Central European humans, we compare and characterize the regions identified by these methods, highlighting four that provide diverse information about autosomes and distinct genomic regions. ANDES is implemented as an open-source package available at https://github.com/riakanjilal/ANDES (accessed on 19 March 2024) and is broadly applicable to genomic data from both model and non-model organisms.

## 2. Materials and Methods

In this section, we begin by describing how genomic variation was pre-processed and summarized into a set of summary statistics. We then outline two approaches for extracting features from these statistics: one based on computing moments of their distributions within genomic windows and another using functional data analysis to model their genomic autocovariation. Next, we detail the distance-based and machine learning models used to detect genomic anomalies from these features, explain how statistical significance is assigned to identify outliers, and conclude with a description of the steps taken in our empirical analysis.

### 2.1. Data Preprocessing and Computation of Summary Statistics

Most contemporary genomic datasets consist of unphased genotype calls for numerous SNPs across the genome. To enable broad applicability across study systems, we used unphased multilocus genotypes (MLGs) derived from biallelic SNPs to assess patterns of genetic variation [62,63,64]. Specifically, we represented each individual’s genotype at an SNP as the number of alternate alleles and used these values to compute a set of eight summary statistics within sliding windows of *ℓ* SNPs. These statistics captured the frequencies of common MLGs and the moments of the distribution of pairwise allele-sharing differences among individuals [65,66]. To characterize spatial patterns in these statistics, we applied two complementary feature extraction strategies: (1) looking at moments summarizing their distribution across *w* consecutive windows and (2) functional data analysis (FDA) to model autocovariation. The resulting features were used as input to unsupervised anomaly detection algorithms designed to identify genomic regions potentially influenced by biological processes or technical artifacts.

We considered only genotypes at biallelic SNPs, coding the genotype of individual i∈{1,2,…,n} on autosome j∈{1,2,…,22} at SNP $k\in \{1,2,\ldots ,L_{j}\}$ as the observed number of copies of the alternate allele $g_{ijk}\in \left\{0,1,2\right\}$ and collected these genotypes in the $n\times L_{j}$-dimensional matrix:

(1) $$G_{j}=\left[\begin{array}{cccc} g_{1j1} & g_{1j2} & \ldots & g_{1jL_{j}}\\ g_{2j1} & g_{2j2} & \ldots & g_{2jL_{j}}\\ \vdots & \vdots & \ddots & \vdots \\ g_{nj1} & g_{nj2} & \ldots & g_{njL_{j}} \end{array}\right].$$

For autosome *j*, we extracted a window of ℓ=51 contiguous SNPs spanning SNP *k* to k+ℓ−1 to create the n×ℓ-dimensional submatrix:

(2) $$G_{jk}=\left[\begin{array}{cccc} g_{1jk} & g_{1j(k+1)} & \ldots & g_{1j(k+\ell -1)}\\ g_{2jk} & g_{2j(k+1)} & \ldots & g_{2j(k+\ell -1)}\\ \vdots & \vdots & \ddots & \vdots \\ g_{njk} & g_{nj(k+1)} & \ldots & g_{nj(k+\ell -1)} \end{array}\right],$$

with windows shifted by a stride of one SNP for all $k\in \{1,2,\ldots ,L_{j}-\ell +1\}$.

We next computed m=8 summary statistics from $G_{jk}$ that captured properties of the distribution of MLG diversity across the *n* sampled individuals in the window of *ℓ* SNPs. Specifically, for each of the n(n−1)/2 distinct pairs of individuals *i* and $i^{\prime }$ (rows of $G_{jk}$), we calculated the Manhattan distance [67]:

(3) $$d\left(i,i^{\prime }\right)=\frac{1}{\ell }\sum_{t=k}^{k+\ell -1}\left|g_{ijt}-g_{i^{\prime }jt}\right|,$$

which quantified the difference between their MLG strings, scaled by the number of SNPs in the window. We then summarized the distribution of these distances, $\left\{d\left(i,i^{\prime }\right):i,i^{\prime }=1,2,\ldots ,n\;\mathrm{and}\;i<i^{\prime }\right\}$, by computing the mean, variance, skewness, and kurtosis, denoted as $\mu_{jk}$, $\sigma_{jk}^{2}$, $\gamma_{jk}$, and $\beta_{jk}$, respectively. In addition, we computed the frequencies of the four most common MLGs across the *n* individuals, denoted as $f_{1,jk}$, $f_{2,jk}$, $f_{3,jk}$, and $f_{4,jk}$, respectively. We selected these summary statistics to capture distortions in MLG spectra and moments of MLG similarity distributions, both of which have proven to be useful in distinguishing between evolutionary events in supervised learning contexts [39,63]. These statistics represent key properties of genomic variation while remaining agnostic to the specific evolutionary processes generating them.

We collected these *m* summary statistics into the m=8-dimensional column vector:

(4) $$S_{jk}=\left[\mu_{jk},\sigma_{jk}^{2},\gamma_{jk},\beta_{jk},f_{1,jk},f_{2,jk},f_{3,jk},f_{4,jk}\right],$$

and stacked these vectors as rows across the $L_{j}-\ell +1$ windows of autosome *j* into the $(L_{j}-\ell +1)\times m$-dimensional matrix:

(5) $$S_{j}=\left[S_{j1}^{T},S_{j2}^{T},\ldots ,S_{j(L_{j}-\ell +1)}^{T}\right],$$

where superscript *T* denotes transpose.

### 2.2. Feature Generation from Summary Statistics

To extract features from the set of *m* summary statistics for autosome *j*, we selected a stretch of w=129 consecutive window locations spanning entries of $S_{j}$ (Equation (4)) from *k* to k+w−1 to create the w×m-dimensional submatrix:

(6) $$S_{jk}=\left[S_{jk}^{T},S_{j(k+1)}^{T},\ldots ,S_{j(k+w-1)}^{T}\right],$$

and considered all $k\in \{1,2,\ldots ,L_{j}-\ell -w+2\}$, with computations shifted by a stride of one window. We then generated features from $S_{jk}$ (Equation (6)) using two strategies.

The first feature generation strategy was to compute moments for each of the m=8 summary statistics to capture properties of their distribution across the *w* windows. For each summary statistic, we computed four moments: mean, variance, skewness, and kurtosis. This resulted in a total of p=4m=32 features, which we collected in the *p*-dimensional column vector:

$$X_{jk}^{\mathrm{Moments}}=\left[X_{jk1}^{\mathrm{Moments}},X_{jk2}^{\mathrm{Moments}},\ldots ,X_{jkp}^{\mathrm{Moments}}\right],$$

where $X_{jkt}^{\mathrm{Moments}}$ is the value of feature t∈{1,2,…,p} summarizing diversity across windows *k* to k+w−1 on autosome *j*.

The second strategy was to employ FDA to approximate the functional form of each summary statistic across the stretch of *w* windows using B=10 cubic spline basis functions (Equation (14)). We also evaluated the velocity and acceleration of each approximated function using the first and second derivatives of the basis expansions, each represented by *B* basis functions (see Section 3.1). We selected cubic splines over other basis functions due to their smoothness, local control, numerical stability, and sparsity, which make them ideal for modeling smooth functional data with complex local behavior [57]. We set B=10 as a compromise between capturing the overall shape of each summary statistic and maintaining computational tractability at a genome-wide scale. This procedure resulted in a total of p=3Bm=240 features representing the basis expansion coefficients from the original functions and their first and second derivatives for each summary statistic. These features were collected in the *p*-dimensional column vector

$$X_{jk}^{\mathrm{FDA}}=\left[X_{jk1}^{\mathrm{FDA}},X_{jk2}^{\mathrm{FDA}},\ldots ,X_{jkp}^{\mathrm{FDA}}\right],$$

where $X_{jkt}^{\mathrm{FDA}}$ is the value of feature t∈{1,2,…,p}, representing the contribution of a basis function to explaining autocovariation patterns in diversity across windows *k* to k+w−1 on autosome *j*.

For each strategy, we assembled the extracted features on autosome *j* in the $(L_{j}-\ell -w+2)\times p$-dimensional matrix:

$$X_{j}^{\mathrm{Method}}=\left[{\left(X_{j1}^{\mathrm{Method}}\right)}^{T},{\left(X_{j2}^{\mathrm{Method}}\right)}^{T},\ldots ,{\left(X_{j(L_{j}-\ell -w+2)}^{\mathrm{Method}}\right)}^{T}\right],$$

and combined the feature matrices across all 22 autosomes into a single N×p-dimensional matrix:

(7) $$X_{\mathrm{Method}}=\left[X_{1}^{\mathrm{Method}},X_{2}^{\mathrm{Method}},\ldots ,X_{22}^{\mathrm{Method}}\right],$$

where $N=\sum_{j=1}^{22}L_{j}-22\left(\ell +w-2\right)$ is the total number of observed window stretches. The variable method indicated whether the features were derived from the moments or FDA strategy.

### 2.3. Construction of Anomaly Detection Algorithms

We applied three anomaly detection algorithms to each of the two feature sets (see Section 2.2), yielding six distinct techniques for outlier identification. Each algorithm took as input the N×p-dimensional matrix $X_{\mathrm{Method}}$ (Equation (7)), where *N* is the number of observations and *p* is the number of features, and output a set of anomaly scores. One method, Mahalanobis distance (MD), computed scores directly as $-\log_{10}$ (*p*-value) from the input feature matrix. The other two methods, isolation forest (IF) and one-class support vector machine (SVM), produced raw anomaly scores, which we then transformed into $-\log_{10}$ (*p*-value) by applying MD to their score distributions. We chose IF and SVM for their complementary strengths: IF offers scalability and low computational cost in high dimensions, while SVM is well-suited to detecting nonlinear structure in data [68,69].

IF identifies anomalies by constructing an ensemble of randomly partitioned isolation trees and flagging observations with short average path lengths across these trees. Two parameters govern this process: the number of trees and the sub-sampling size used to build each one. During tree construction, observations are recursively partitioned by randomly selecting a feature and a split value. Because anomalous observations are both rare and distinct, they tend to be isolated in fewer steps, resulting in shorter path lengths. Thus, a low average path length across the forest indicates a high likelihood of being an anomaly [68].

To elaborate, each isolation tree recursively divides the dataset until all observations are isolated in terminal nodes (leaves). Assuming that all observations are distinct, a fully grown tree will contain *N* external nodes and N−1 internal nodes. For each observation, the anomaly score is inversely related to the average number of edges traversed to isolate it, aggregated across all trees in the forest. The shorter this path, the more likely it is that the observation is anomalous.

In contrast, an SVM defines a decision boundary that separates typical from anomalous observations by learning an affine function characterized by an intercept $\theta_{0}$ and a *p*-dimensional coefficient vector θ. These coefficients represent the relative importance of each feature in defining the boundary. To capture nonlinear relationships, the algorithm applies a feature map ϕ:X→ϕ(X) that projects the data into a higher-dimensional space, where it learns a linear decision boundary of the form $\left\{x\;|\;\theta_{0}+\theta^{T}\phi \left(x\right)=0\right\}$ [70,71]. In this space, each observation *X* is assigned a label via $\mathrm{sign}(\theta_{0}+\theta^{T}\phi \left(X\right))$, where sign(x) equals 1 if x>0, 0 if x=0, and −1 if x<0. This approach allows the SVM to distinguish anomalous data points that deviate from the bulk of the distribution, even when these deviations are subtle or nonlinear.

### 2.4. Identification of Anomalous Regions

Anomaly scores produced by the IF and SVM models were log-transformed to normalize and increase the spread of their distributions. For each method, we computed squared Mahalanobis distances across the *N* observations. For the IF and SVM methods, each observation was represented by a single score, so the number of features used was p=1. In particular, for a given observation encoded as a *p*-dimensional column vector, the squared Mahalanobis distance was defined as

$$D^{2}={\left(X-\bar{X}\right)}^{T}C^{-1}\left(X-\bar{X}\right),$$

where $\bar{X}$ is the sample mean across observations and C is the p×p sample covariance matrix [72].

These distances follow a Hotelling’s *T*-squared distribution [73,74], which can be transformed to an *F* distribution with degrees of freedom *p* and N−p using the relation [75]

(8) $$F_{p,N-p}=\frac{N-p}{p(N-1)}D^{2}.$$

We used this expression to compute *p*-values for each observation.

However, quantile–quantile plots revealed inflation in the distribution of *p*-values relative to the expected uniform distribution [76]. To correct this issue, we applied a linear regression approach [77] to estimate an inflation factor λ. Specifically, we fit a linear regression model through the origin to predict the $\chi^{2}$ quantile function computed for our unadjusted *p*-values from the $\chi^{2}$ quantiles derived from a set of uniform probabilities. We then divided the uncorrected $\chi^{2}$ quantiles by λ and converted these adjusted values into *p*-values. To account for multiple testing, we applied a Bonferroni correction [78] with a significance threshold of $\alpha =0.05/10^{6}=5\times 10^{-8}$, where the denominator accounts for approximately one million independent loci in the human genome, as is widely used in association studies [79]. Observations with *p*-values below this threshold were considered outliers.

To visualize genomic variation at the MHC locus, we generated images of MLG diversity at outlier windows associated with HLA genes. To plot the image of MLG diversity for a given outlier data point, we examined the consecutive ℓ+w−1 SNPs that defined the data point and collected the genotypes into the n×(w+ℓ−1)-dimensional matrix:

(9) $$G=\left[\begin{array}{cccc} g_{11} & g_{12} & \ldots & g_{1(w+\ell -1)}\\ g_{21} & g_{22} & \ldots & g_{2(w+\ell -1)}\\ \vdots & \vdots & \ddots & \vdots \\ g_{n1} & g_{n2} & \ldots & g_{n(w+\ell -1)} \end{array}\right],$$

where $g_{ij}\in \left\{0,1,2\right\}$ denotes the number of minor alleles for individual i∈{1,2,…,n} at SNP j∈{1,2,…,w+ℓ−1}. From this matrix, we extracted *ℓ*-SNP windows from positions *k* to k+ℓ−1 and generated submatrices for all k∈{1,2,…,w}, with a stride of one SNP, which we denote as

(10) $$G_{k}=\left[\begin{array}{cccc} g_{1k} & g_{1(k+1)} & \ldots & g_{1(k+\ell -1)}\\ g_{2k} & g_{2(k+1)} & \ldots & g_{2(k+\ell -1)}\\ \vdots & \vdots & \ddots & \vdots \\ g_{nk} & g_{n(k+1)} & \ldots & g_{n(k+\ell -1)} \end{array}\right].$$

We sorted the rows of each $G_{k}$ in ascending order by their $L_{1}$-norm and denoted the sorted matrix as

(11) $$G_{k}^{\mathrm{Sort}}=\left[\begin{array}{cccc} g_{1k}^{\mathrm{Sort}} & g_{1(k+1)}^{\mathrm{Sort}} & \ldots & g_{1(k+\ell -1)}^{\mathrm{Sort}}\\ g_{2k}^{\mathrm{Sort}} & g_{2(k+1)}^{\mathrm{Sort}} & \ldots & g_{2(k+\ell -1)}^{\mathrm{Sort}}\\ \vdots & \vdots & \ddots & \vdots \\ g_{nk}^{\mathrm{Sort}} & g_{n(k+1)}^{\mathrm{Sort}} & \ldots & g_{n(k+\ell -1)}^{\mathrm{Sort}} \end{array}\right].$$

Let $I_{j}\left(k\right)\in \left\{0,1\right\}$ be an indicator variable denoting whether SNP *j* is present in $G_{k}^{\mathrm{Sort}}$ and let $\psi_{j}\left(k\right)\in \left\{1,2,\ldots ,\ell \right\}$ denote its corresponding column index if present. We then defined a n×(w+ℓ−1) data matrix of the form

(12) $$X=\left[\begin{array}{cccc} x_{11} & x_{12} & \ldots & x_{1(w+\ell -1)}\\ x_{21} & x_{22} & \ldots & x_{2(w+\ell -1)}\\ \vdots & \vdots & \ddots & \vdots \\ x_{n1} & x_{n2} & \ldots & x_{n(w+\ell -1)} \end{array}\right],$$

where

(13) $$x_{ij}=\frac{\sum_{k=1}^{w}g_{i\psi_{j}\left(k\right)}^{\mathrm{Sort}}\cdot I_{j}\left(k\right)}{\sum_{k=1}^{w}I_{j}\left(k\right)}$$

is the mean allele count for individual *i* across all windows that include SNP *j*. This final matrix X was used to generate images of local MLG diversity at outlier regions.

### 2.5. Application of Methods to Empirical Data

We applied ANDES to autosomal genotype data from 99 individuals of Central European ancestry (CEU) from the 1000 Genomes Project [80]. First, we generated MLGs from biallelic SNP data using the allel module from the scikit-allel package [81] and computed summary statistics using the stat and special.distance modules from the SciPy library [82] in Python [83]. Next, we computed moment and FDA features. Moment features were calculated in Python using the stat module of SciPy [82,83], whereas FDA features were calculated in R [84] using the methodology outlined by [85], implemented via the fda package [86]. For the MD-M and MD-F methods, anomaly scores were computed directly from moment and FDA features using the mahalanobis function in the MASS package [87] of R [84]. For IF-M and IF-F, we trained isolation forests with default parameters using the IsolationForest function in the Scikit-Learn library [88] and then computed anomaly scores for all observations using the decision_function function. These scores were transformed into $-\log_{10}$ (*p*-value) using the mahalanobis function from the MASS package [87].

Similarly, for SVM-M and SVM-F, we trained one-class SVMs using the OneClassSVM function from Scikit-Learn library [88] in Python [83], with parameters kernel= ‘rbf’ and gamma = ‘auto’. Due to the poor scalability of SVMs with large sample sizes, we employed mini-batch training and systematic sampling. Because SVM models scale poorly (at least quadratically) with the number of input samples, we selected 18 large mini-batches—distributed across 22 chromosomes—to maximize batch size while ensuring that model training remained computationally tractable. Specifically, we partitioned each feature matrix $X_{\mathrm{Method}}$ into m=18 non-overlapping subsets through systematic sampling within each subset. To mitigate correlations between observations across subsets, we started at the *i*th observation, where i∈{1,2,…,m}, and then selected every 10th observation thereafter, yielding 18 mini-batches for training the SVM. After model training, we used the score_sample function to compute anomaly scores, which were transformed to $-\log_{10}$ (*p*-values) using the mahalanobis function in R [84]. From the six methods implemented in ANDES, we selected four (MD-M, MD-F, IF-M, and IF-F) for downstream analyses based on their tendency to produce orthogonal outlier patterns.

To evaluate associations of outliers with biologically-relevant genomic regions, we intersected locations of the *N* observations with annotations from the “RefSeq”, “knownGene”, and “gc5Base” tables of the hg19 reference genome via the UCSC Genome Browser [89]. These annotations were used to classify outliers as intergenic or genic, and further assign genic regions to exons, introns, 5′UTRs, and 3′UTRs, considering only the longest transcript per gene. Similarly, we also investigated associations of outliers with technical artifacts by intersecting their locations with repetitive regions from the “fa.masked” table and extracting alignability and mappability scores from the CRG 100mer track [90] from the UCSC Genome Browser [89]. Regions with CRG scores ≤ 0.9 were considered low-confidence due to poor alignability or mappability [91].

### 2.6. Statistical Analyses

All statistical analyses were performed using the stat module from the SciPy library [82] in Python. Multinomial tests were used to compare observed distributions of outlier windows to those expected under a uniform distribution and evaluate whether observed outlier windows were uniformly distributed across 22 autosomes and four regions of protein-coding genes (exons, introns, 5′UTRs, and 3′UTRs). Two-tailed binomial tests were employed to compare observed and expected numbers of outlier windows and evaluate their over- and underrepresentations on individual chromosomes, in intergenic regions, in each of the four regions of protein-coding genes, in regions with low GC content, in repetitive regions, and in regions with low CRG (mappability and alignability) scores. For each binomial test, we set the number of successes *x* as the number of observed outlier windows in the region of interest, the number of trials *n* as the total number of outlier windows, and the probability of success *p* as the expected proportion of outlier windows in that region.

Because linkage disequilibrium introduces correlations among adjacent genomic windows, the assumption of statistical independence required for these tests may be violated, potentially inflating significance values. To address this issue, we performed $10^{4}$ permutations for each test: shuffling chromosome labels for chromosome-wise analyses and region labels for region-wise analyses. These permutations produced a null distribution of *p*-values for each anomaly detection method. For each original test, we calculated the fraction of permuted *p*-values that was smaller than the observed *p*-value, referring to this fraction as the permutation *p*-value. We considered results significant if this value was below a defined threshold α. We set α=0.05 for multinomial tests. For binomial tests, we utilized the Bonferroni-corrected thresholds of $\alpha =0.05/22=2.27\times 10^{-3}$ for chromosome-wise analyses, $\alpha =0.05/4=1.25\times 10^{-2}$ for protein-coding region-wise analyses with four labels, and $\alpha =0.05/2=2.50\times 10^{-2}$ for all genomic region-wise analyses with two labels.

### 2.7. Gene Ontology Enrichment Analyses

We performed Gene Ontology (GO) enrichment analyses to assess functional enrichment in genes with high anomaly scores using the web-based GOrilla tool at https://cbl-gorilla.cs.technion.ac.il/ [92,93] (accessed on 16 February 2023). In particular, genes were ranked by anomaly score for each of the four methods (Supplementary Tables S11–S14) and then used as input to GOrilla, which searches for enriched GO terms that appear densely at the top of a ranked list of genes [92,93]. For each run, we chose “Homo sapiens” as the organism, set the running mode to “Single ranked list of genes”, and selected all ontologies (process, function, and component). To account for multiple testing, we only considered terms as significantly enriched if their false discovery rate *q*-value <0.05.

## 3. Results

### 3.1. Design of Anomaly Detection Algorithms

To detect regions of the genome with unusual patterns of variation, we began by computing eight summary statistics from MLGs across sliding windows of *ℓ* SNPs. These statistics included the frequencies of the four most common MLGs, as well as the mean (central tendency), variance (spread), skewness (asymmetry), and kurtosis (tail weight) of pairwise allele-sharing differences among individuals [65,66]. To capture spatial structure, we analyzed how these statistics varied across *w* consecutive windows, centered at each focal window.

We extracted features from these statistics using two strategies. First, for each of the eight statistics, we computed its mean, variance, skewness, and kurtosis across the *w* windows, yielding 32 moment-based features. Second, we used FDA to model the spatial patterns of each statistic. Specifically, we treated the statistic as a function of genomic position and approximated it using a linear combination of B=10 cubic spline basis functions:

(14) $$f\left(t\right)\approx \sum_{b=1}^{B}c_{b}\;\phi_{b}\left(t\right),$$

where $\phi_{b}\left(t\right)$ is the *b*th univariate basis function and $c_{b}$ is the associated *b*th basis coefficient that provides the degree that $\phi_{b}\left(t\right)$ contributes to f(t). To enrich this representation, we also included coefficients from the first and second derivatives of each function, capturing the local “velocity” and “acceleration” of change. This yielded 3B features per statistic or 240 total FDA-based features. All features were assigned to the center position of their corresponding *w*-window stretch.

**Figure 1:**
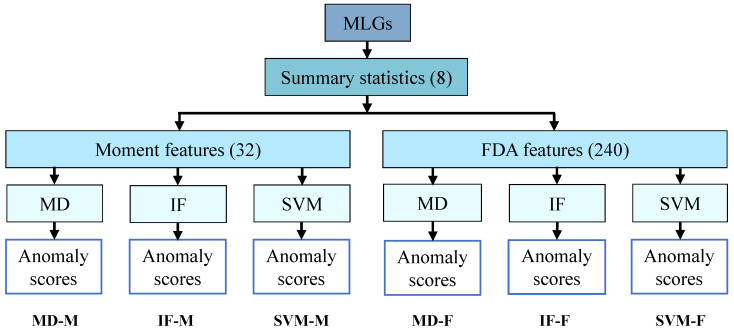
Schematic overview of the six ANDES methods for anomaly detection. MLGs were used to compute summary statistics, which were then transformed into sets of moment and FDA features. Each feature set was analyzed using three anomaly detection algorithms, yielding six distinct sets of anomaly scores.

To identify anomalous regions, we applied three unsupervised anomaly detection algorithms to each of the two feature sets, generating six distinct methods (Figure 1). Briefly, MLGs were used to compute eight summary statistics, from which the two sets of features were extracted to implement anomaly detection. Each feature set was then analyzed using three algorithms, yielding anomaly scores that we transformed into *p*-values (see Section 2). We considered both distance-based [94] and machine learning [68,95] approaches. For the distance-based approach, we calculated Mahalanobis distances (MDs) [72,96] across the full set of features for all genomic regions and derived *p*-values from these distances. We refer to these methods as MD-M and MD-F, denoting MD-based anomaly detection applied to moment and FDA features, respectively. For the machine learning-based approaches, we used two widely adopted algorithms: isolation forest (IF) [68] and one-class support vector machine (SVM) [95]. Both generate anomaly scores, which we converted to *p*-values to allow for consistent thresholding across all methods. We refer to the IF-based methods using moment and FDA features as IF-M and IF-F, respectively, and the SVM-based methods as SVM-M and SVM-F.

### 3.2. Comparison of Anomaly Detection Methods

As a proof of concept, we applied ANDES to autosomal genotype calls from 99 individuals of Central European ancestry (CEU) from the 1000 Genomes Project dataset [80]. We considered only biallelic SNPs and encoded genotypes as counts (zero, one, or two) of alternate alleles. Within each ℓ=51 SNP window, MLGs were formed as strings of these values and used to compute summary statistics, which then served as the basis for feature construction and input to ANDES.

**Figure 2:**
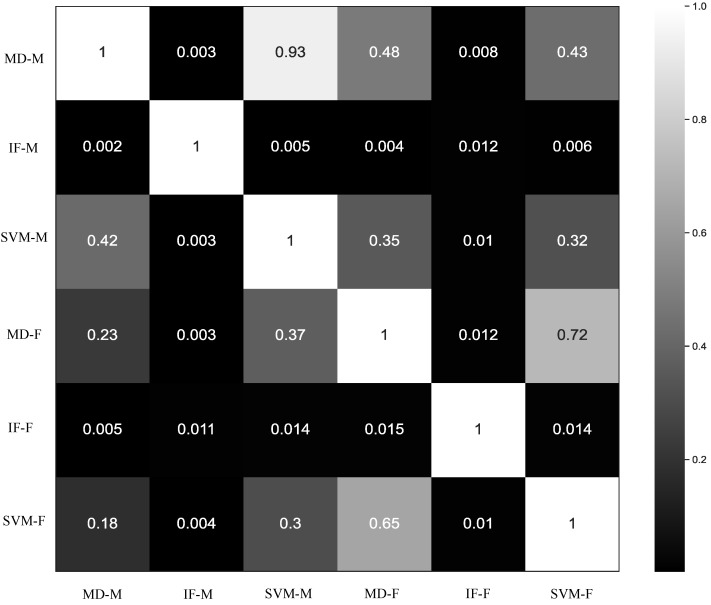
Heatmaps showing the overlap of significant outlier windows across the six ANDES methods. Each cell indicates the fraction of outlier windows identified by one method (row *i*) that were also detected by another method (column *j*), producing an asymmetric matrix. Higher values reflect greater agreement between methods, helping to visualize their relative similarity or distinctiveness.

To assess how the six ANDES methods differed in practice, we compared overlap among the genomic windows flagged as significant outliers (Figure 2). We found that outlier windows detected by methods based on moment features were generally not recapitulated by those based on FDA features, and vice versa. Among methods using the same feature type, IF-based methods often detected distinct windows compared to MD- or SVM-based approaches. In contrast, MD and SVM tended to identify similar sets of outliers. Based on these observations, we selected MD-M, MD-F, IF-M, and IF-F for further analyses due to their relatively orthogonal behavior and potential to capture distinct genomic signatures. Using a genome-wide significance threshold of $\alpha =5\times 10^{-8}$ (see Section 2), these four methods detected 8291 (MD-M), 11,808 (IF-M), 17,468 (MD-F), and 13,590 (IF-F) outlier windows.

**Figure 3:**
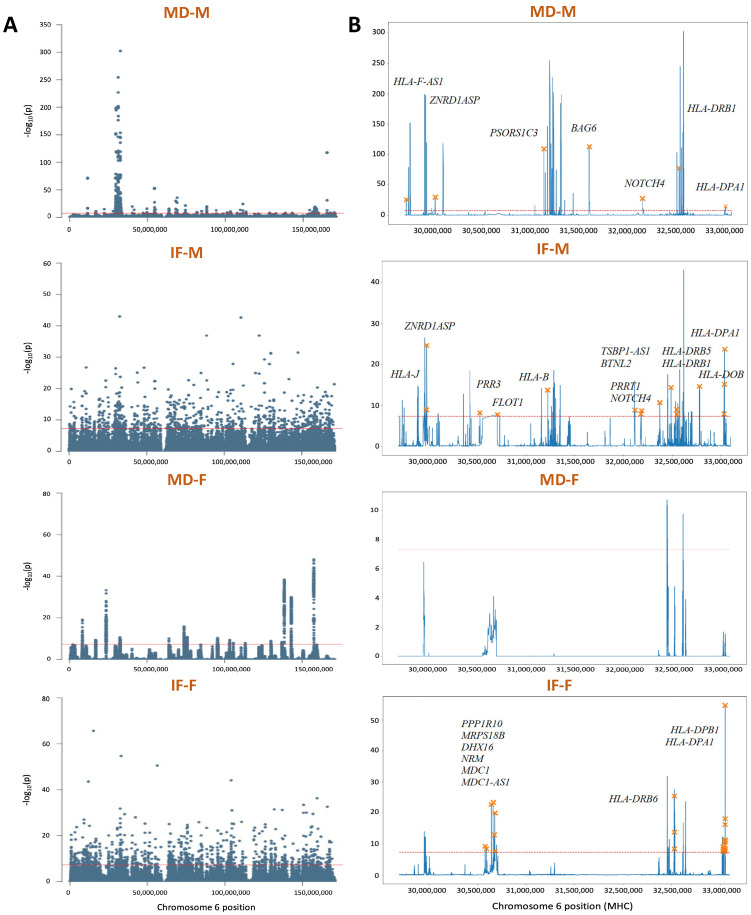
Manhattan plots of anomaly scores for (**A**) chromosome 6 and (**B**) the MHC region on chromosome 6 for the MD-M, IF-M, MD-F, and IF-F methods. The *x*-axis denotes the center positions of windows, the horizontal red line marks the genome-wide significance threshold ($\alpha =5\times 10^{-8}$), and orange crosses indicate peaks associated with specific labeled genes.

We next visualized the genome-wide distributions of anomaly scores from each of the four selected methods (Supplementary Figure S1). All four methods produced significant peaks across the genome, with sharper and more defined peaks for the MD-based methods. Consistent with this observation, there were fewer peaks for MD-based methods, with totals of 777, 8470, 792, and 10,560 peaks for the MD-M, IF-M, MD-F, and IF-F methods, respectively. Notably, all four methods harbored a high density of peaks on chromosome 6 (Figure 3A), with MD-M displaying one particularly large cluster of peaks at the major histocompatibility complex (MHC) locus that is thought to have undergone natural selection in humans [97,98]. Zooming into this region, the four methods shared a number of isolated peaks (Figure 3B), with several corresponding to the human leukocyte antigen (HLA) genes previously associated with balancing selection, including *HLA-B*, *HLA-DRB1*, *HLA-DRB5*, *HLA-DPA1*, *HLA-DPB1*, and *HLA-DOB* [99,100,101].

**Figure 4:**
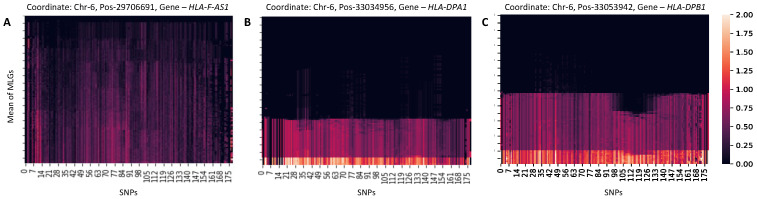
Images of MLG diversity of top outlier windows associated with HLA genes for (**A**) MD-M, (**B**) IF-M, and (**C**) IF-F. MD-F did not identify any HLA-associated outliers. Each image displays mean values of sorted genotype matrices ($G_{k}^{\mathrm{Sort}}$; see Section 2), with rows representing MLGs and columns representing SNPs across all windows k∈{1,2,…,w}. Pixel values range from zero (darkest, homozygous major allele) to two (brightest, homozygous minor allele), with intermediate shades indicating high frequencies of heterozygous genotypes. MLGs are sorted top to bottom by mean genotype value, so rows near the top generally have more homozygous major alleles. “Coordinate” labels indicate the chromosome, position, and gene associated with each outlier window.

To examine these outlier signals more closely, we generated images of MLG diversity for top outlier windows near HLA genes (Figure 4, see Section 2). These plots revealed distinctive patterns of reduced diversity. In particular, the window flagged by MD-M at the *HLA-F-AS1* gene was characterized by high frequencies of heterozygous genotypes (Figure 4A), while the windows flagged by IF-M at the *HLA-DPA1* gene and IF-F at the *HLA-DPB1* gene both displayed intermediate frequencies of homozygous major alleles and heterozygous genotypes that were accompanied by low frequencies of homozygous minor alleles (Figure 4B,C). Such patterns may reflect past balancing selection, which can maintain genetic diversity by favoring heterozygosity or preserving multiple alleles at a locus.

### 3.3. Characterization of Anomalous Regions

The goal of ANDES was to identify anomalous regions affected by biological phenomena or technical artifacts. Because such regions were expected to be non-uniformly distributed across the genome, we first compared the observed numbers of outlier windows on each chromosome to those expected under a uniform distribution. For all four selected methods, outlier windows deviated significantly from uniformity, with consistent overrepresentations on chromosome 4 (Supplementary Table S1). Similarly, we observed significant overrepresentations of outlier windows in intergenic regions for all methods (Supplementary Table S2). To investigate the biological relevance of outlier windows, we also compared their distributions across exons, introns, and 5′ and 3′ untranslated regions (UTRs) of protein-coding genes. Consistent with our other findings, outlier windows were non-uniformly distributed across genic regions, with overrepresentations in introns for three of the methods (Supplementary Table S3). Outlier windows were also enriched in regions with low GC content for all methods (Supplementary Table S4), repetitive regions for all methods (Supplementary Table S5), and low CRG alignability and mappability scores for three methods (Supplementary Table S6). Filtering regions with repeats and low CRG scores did not substantially alter genomic region-wise distributions or low GC content of resulting outlier windows (Supplementary Tables S7–S10; see Section 2).

To gain insight into the biological processes associated with outlier genes, we performed Gene Ontology (GO) enrichment analyses for each method using ranked lists of protein-coding genes, where rankings were based on the minimum *p*-value across associated outlier windows (Supplementary Tables S11–S14; see Section 2). These analyses uncovered many of the same enriched GO terms across all four methods. Within the cellular component domain, the common terms were “neuron part”, “cell projection”, “synapse part”, “plasma membrane”, and “membrane” (Supplementary Tables S15–S18). For the biological processes domain, “cell adhesion” and “biological adhesion” were consistently enriched (Supplementary Tables S19–S22). The molecular function domain yielded a broader set of shared terms, including “ion binding”, “ion transmembrane transporter activity”, “ion channel activity”, “channel activity”, “substrate-specific channel activity”, “gated channel activity”, “cation channel activity”, “passive transmembrane transporter activity”, “metal ion transmembrane transporter activity”, “inorganic molecular entity transmembrane transporter activity”, “ATP-dependent microtubule motor activity, minus-end-directed”, “adenylate cyclase inhibiting G protein-coupled glutamate receptor activity”, “G protein-coupled glutamate receptor activity”, “ATPase activity, coupled”, “cell adhesion molecule binding”, “cyclic-nucleotide phosphodiesterase activity”, “3’,5’-cyclic-nucleotide phosphodiesterase activity”, “ATP binding”, and “actin binding” (Supplementary Tables S23–S26). Taken together, these results suggest that top-ranking outlier genes identified by ANDES are often involved in neuronal processes, particularly those related to nervous system development and neuronal signaling.

While the enriched GO categories were broadly consistent, the identities of top-ranking genes varied among methods. The MD-M method ranked *XIRP2* highest, which encodes an actin-binding protein that stabilizes actin filaments. The IF-M method identified *DNAH9*, a gene encoding a dynein heavy chain involved in the movement of cilia and flagella. For the MD-F method, the top gene was *SEMA6C*, which encodes a signaling molecule implicated in the cellular response following central nervous system injury. The IF-F method prioritized *VAV2*, a guanine nucleotide exchange factor for Rho GTPases that regulates actin cytoskeleton dynamics. Among these genes, *DNAH9* has shown evidence of positive selection in several non-human mammals [102,103,104,105,106,107], while *VAV2* has been identified as a target of selection in the Yoruba population [108]. Despite variation in the specific genes flagged by each method, correlations in gene rankings aligned with broader patterns observed across methods (Figure 2). The strongest correlations occurred between methods using the same set of features (ρ=0.68 for MD-M and IF-M; ρ=0.63 for MD-F and IF-F), followed by those using the same anomaly detection algorithm (ρ=0.52 for MD-M and MD-F; ρ=0.55 for IF-M and IF-F), and, lastly, by those using different features and algorithms (ρ=0.51 for MD-M and IF-F; ρ=0.50 for MD-F and IF-M).

## 4. Discussion

In this study, we presented ANDES, a suite of anomaly detection methods for identifying genomic regions that exhibit aberrant patterns of genetic variation due to biological or technical factors. ANDES detects such regions using unsupervised anomaly detection models trained on statistical features extracted from summaries of genetic variation. Importantly, ANDES explicitly accounts for expected autocovariation in genetic diversity caused by linkage disequilibrium by incorporating FDA techniques into its feature extraction process. Because it operates on MLGs and does not rely on assumptions about underlying evolutionary processes, ANDES is broadly applicable across both model and non-model systems for detecting anomalous genomic regions, regardless of their source.

Within the ANDES framework, we implemented both distance-based (MD) and machine learning-based (IF and SVM) anomaly detection algorithms, each offering distinct advantages. Distance-based approaches are conceptually straightforward and computationally efficient, making them suitable for large datasets. In particular, the scale-invariance property of MD ensures that features contribute proportionally, even if they differ in scale, and this approach also effectively incorporates correlations among features [109,110]. Additionally, MD enables the direct computation of *p*-values, enabling the uniform application of significance thresholds for anomaly detection. Notably, most anomaly scores generated by MD-F yielded *p*-values close to one, suggesting that FDA effectively captures local genomic trends by modeling expected autocovariation under linkage disequilibrium, which may explain its identification of fewer peaks than MD-M in our empirical analysis. In contrast, machine learning algorithms were selected for their performance with unlabeled data and adaptability to complex, nonlinear patterns. IF, in particular, scales well to large datasets and can efficiently isolate anomalies, making it more computationally tractable than an SVM. In light of these findings, we recommend MD-F as the default method when applying ANDES due to its ability to capture genomic correlations induced by linkage disequilibrium, computational efficiency, and capacity to directly compute *p*-values with a distribution appropriately skewed toward non-significant values (i.e., non-outliers).

The application of ANDES to European human genomes uncovered numerous anomalous peaks within the MHC region (Figure 3B), a locus known to be affected by both biological and technical factors. Specifically, the MHC locus harbors many genes that underwent balancing selection in humans [99,100,101], and our results corroborate this, identifying several peaks in this region (Figure 4). These signals may reflect the persistence of multiple alleles maintained by balancing selection, resulting in elevated heterozygosity [111]. The MHC locus is also ridden with structural variation, complicating genome assembly [112] and potentially introducing variant calling, genotyping, and phasing errors. Thus, the density of peaks in this region likely results from a combination of biological processes and technical artifacts, both of which are important to consider in evolutionary and biomedical studies.

Our characterization of anomalous regions revealed that outlier windows were non-uniformly distributed across the genome, with overreprentations on specific autosomes, in intergenic relative to genic regions, in introns compared to other regions of protein-coding genes, and in regions with low GC content, repetitive elements, or poor mappability (Supplementary Tables S1 and S6). Removing repetitive and low mappability regions did not substantially alter these patterns (Supplementary Tables S7–S10), suggesting that these technical factors did not drive the overall distributions of outlier windows. Prior work suggests that structural variation, which may contribute to both biological signals and technical artifacts, can significantly affect gene function through regulatory mechanisms and adaptation [113]. Moreover, adaptive signals have been shown to correlate more strongly with regulatory than with protein-coding regions [114], consistent with our observation that outlier regions are enriched in noncoding sequences.

Additionally, GO enrichment analyses of protein-coding genes ranked by anomaly scores uncovered enrichments of functions related to neuronal development and signaling (Supplementary Tables S15–S26). These enrichments likely reflect both biological relevance and model sensitivity. Neuronal genes are often regulated by complex networks of enhancers and other elements [115], which can generate intricate patterns of variation. These loci may also be subject to recent selective pressures linked to behavioral, cognitive, or sensory traits in humans. Because ANDES operates without assuming specific evolutionary mechanisms, it may be particularly sensitive to detecting such complex regulatory architectures. Further, though gene rankings were highly correlated among methods, top-ranked genes varied, with two previously identified as targets of positive selection [102,103,104,105,106,107,108]. Collectively, these results showcase the ability of ANDES to pinpoint anomalous genomic regions associated with diverse biological phenomena or technical artifacts, providing a valuable tool for dissecting the evolutionary processes underlying genomic diversity.

## Data Availability

Polymorphism data analyzed in this study are publicly available at http://www.1000genomes.org/ (accessed on 11 July 2022).

## Supplementary Materials

The following supporting information can be downloaded at https://www.mdpi.com/article/10.3390/genes16060710/s1: Figure S1: Manhattan plots of anomaly scores computed using (A) MD-M, (B) IF-M, (C) MD-F, and (D) IF-F; Table S1: Observed and expected outlier windows across autosomes for MD-M, IF-M, MD-F, and IF-F; Figure S2: Plots depicting top peaks identified by (A) MD-M, (B) IF-M, (C) MD-F, and (D) IF-F; Table S2: Observed and expected outlier windows in intergenic regions for MD-M, IF-M, MD-F, and IF-F; Table S3: Observed and expected outlier windows across four regions of protein-coding genes for MD-M, IF-M, MD-F, and IF-F; Table S4: Observed and expected outlier windows in genomic regions with low GC content (<50%) for MD-M, IF-M, MD-F, and IF-F; Table S5: Observed and expected outlier windows in repetitive regions for MD-M, IF-M, MD-F, and IF-F; Table S6: Observed and expected outlier windows in genomic regions with low CRG scores (≤0.9) for MD-M, IF-M, MD-F, and IF-F; Table S7: Observed and expected outlier windows across autosomes after removing repetitive regions and regions with low CRG scores (≤0.9) for MD-M, IF-M, MD-F, and IF-F; Table S8: Observed and expected outlier windows in intergenic regions after removing repetitive regions and regions with low CRG scores (≤0.9) for MD-M, IF-M, MD-F, and IF-F; Table S9: Observed and expected outlier windows across four regions of protein-coding genes after removing repetitive regions and regions with low CRG scores (≤0.9) for MD-M, IF-M, MD-F, and IF-F; Table S10: Observed and expected outlier windows with low GC content (<50%) after removing repetitive regions and regions with low CRG scores (≤0.9) for MD-M, IF-M, MD-F, and IF-F; Tables S11–S14: Ranked lists of all protein-coding genes utilized as input for the GO analysis for MD-M, IF-M, MD-F, and IF-F, respectively; Tables S15–S18: Identification of statistically significant and enriched GO terms through GO component analysis using ranked lists of all protein-coding genes for MD-M, IF-M, MD-F, and IF-F, respectively; Tables S19–S22: Identification of statistically significant and enriched GO terms through GO process analysis using a ranked list of all protein-coding genes for MD-M, IF-M, MD-F, and IF-F, respectively; Tables S23–S26: Identification of statistically significant and enriched GO terms through GO function analysis using a ranked list of all protein-coding genes for MD-M, IF-M, MD-F, and IF-F, respectively.
