# Supplementary material for: Genomic Anomaly Detection with Functional Data Analysis
Source: Genes (Basel). 2025 Jun 15;16(6):710. doi: 10.3390/genes16060710 (PMC12192579; doi:10.3390/genes16060710)
Supplement: Supplementary file 1 [file genes-16-00710-s001.zip › SupplementaryData1.pdf]

Supplementary material

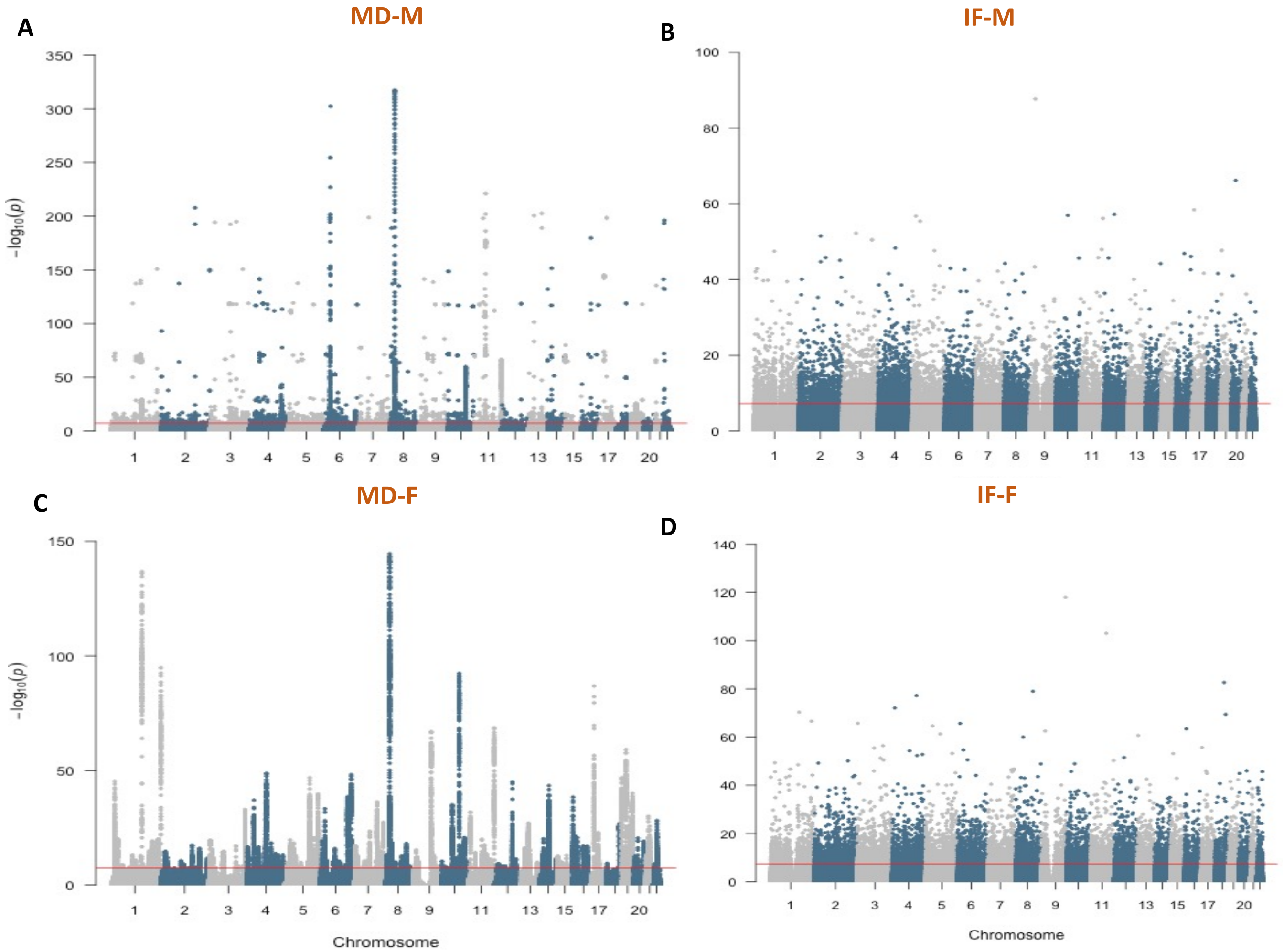

Figure S1: Manhattan plots of anomaly scores computed using (A) MD-M, (B) IF-M, (C) MD-F, and (D) IF-F. Values along x-axes represent center positions of windows on each of the 22 human autosomes. Horizontal red lines denote the genome-wide p-value significance cutoff of $\alpha = 5 \times 10^{-8}$.

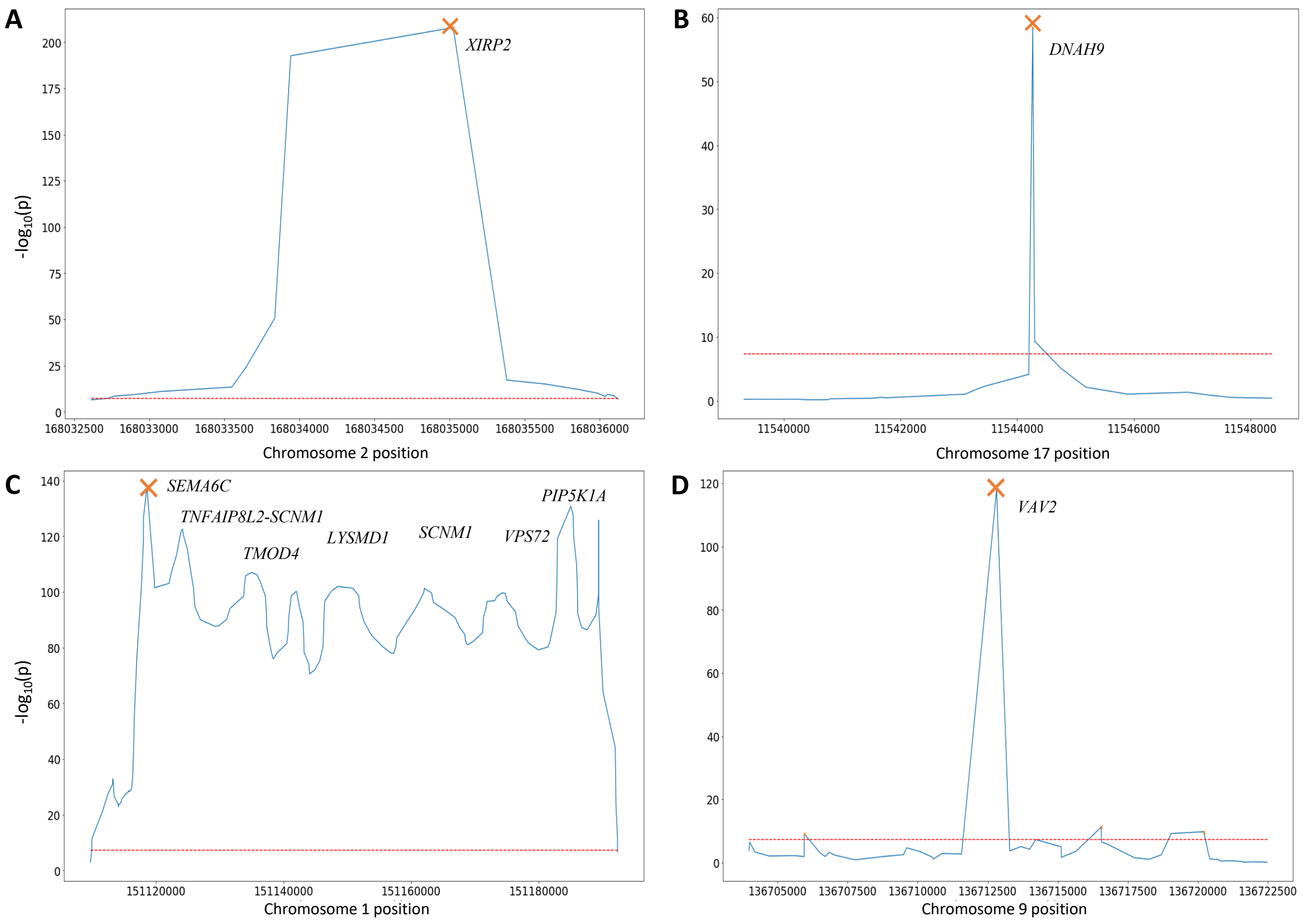

Figure S2: Plots depicting top peaks identified by (A) MD-M, (B) IF-M, (C) MD-F, and (D) IF-F. Each panel displays the highest peak that is associated with a gene, which is indicated by an orange cross. Horizontal red lines denote the genome-wide p-value significance threshold of $\alpha = 5 \times 10^{-8}$.

Table S1: Observed and expected outlier windows across autosomes for MD-M, IF-M, MD-F, and IF-F.

Chr	Observed	Expected	Permutation p-value	Observed	Expected	Permutation p-value
	Method: MD-M			Method: IF-M		
1	766	640.86	$< \mathbf{10^{-4}}$	864	907.02	1.40×10^{-1}
2	180	692.50	$< \mathbf{10^{-4}}$	921	990.36	2.05×10^{-2}
3	402	587.16	$< \mathbf{10^{-4}}$	882	834.20	7.85×10^{-2}
4	761	604.77	$< \mathbf{10^{-4}}$	1,137	851.44	$< \mathbf{10^{-4}}$
5	494	521.70	2.19×10^{-1}	713	742.91	2.61×10^{-1}
6	758	559.75	$< \mathbf{10^{-4}}$	819	798.16	4.38×10^{-1}
7	416	493.15	$\mathbf{3.00 \times 10^{-4}}$	770	699.81	7.50×10^{-3}
8	1,019	458.47	$< \mathbf{10^{-4}}$	658	651.97	7.99×10^{-1}
9	425	360.52	$\mathbf{3.00 \times 10^{-4}}$	433	518.53	$\mathbf{1.00 \times 10^{-4}}$
10	300	424.55	$< \mathbf{10^{-4}}$	644	606.90	1.18×10^{-1}
11	356	410.63	4.90×10^{-3}	514	587.63	$\mathbf{1.30 \times 10^{-3}}$
12	53	394.97	$< \mathbf{10^{-4}}$	578	565.73	5.79×10^{-1}
13	465	304.80	$< \mathbf{10^{-4}}$	406	436.35	1.38×10^{-1}
14	460	270.57	$< \mathbf{10^{-4}}$	335	384.95	9.80×10^{-3}
15	63	236.07	$< \mathbf{10^{-4}}$	298	337.28	2.32×10^{-2}
16	98	258.11	$< \mathbf{10^{-4}}$	355	370.75	4.15×10^{-1}
17	172	222.74	$\mathbf{2.00 \times 10^{-4}}$	299	318.04	2.80×10^{-1}
18	205	236.38	3.89×10^{-2}	359	337.22	2.15×10^{-1}
19	676	196.91	$< \mathbf{10^{-4}}$	297	275.12	1.65×10^{-1}
20	10	182.68	$< \mathbf{10^{-4}}$	204	261.40	$\mathbf{1.00 \times 10^{-4}}$
21	19	116.52	$< \mathbf{10^{-4}}$	169	163.69	6.33×10^{-1}
22	193	117.18	$< \mathbf{10^{-4}}$	153	168.54	2.25×10^{-1}
All			$< \mathbf{10^{-4}}$			$< \mathbf{10^{-4}}$
	Method: MD-F			Method: IF-F		
1	1,434	1,350.92	1.74×10^{-2}	1,050	1,049.70	9.74×10^{-1}
2	543	1,457.52	$< \mathbf{10^{-4}}$	1,066	1,134.68	3.10×10^{-2}
3	696	1,236.68	$< \mathbf{10^{-4}}$	1,044	960.61	5.10×10^{-3}
4	1,492	1,274.59	$< \mathbf{10^{-4}}$	1,070	991.32	$\mathbf{1.80 \times 10^{-3}}$
5	1,380	1,098.32	$< \mathbf{10^{-4}}$	888	852.17	2.02×10^{-1}
6	954	1,181.52	$< \mathbf{10^{-4}}$	875	919.05	1.31×10^{-1}
7	1,457	1,038.00	$< \mathbf{10^{-4}}$	807	805.56	9.58×10^{-1}
8	1,292	966.37	$< \mathbf{10^{-4}}$	795	752.51	1.03×10^{-1}
9	671	759.89	$\mathbf{4.00 \times 10^{-4}}$	528	592.19	7.50×10^{-3}
10	861	893.73	2.63×10^{-1}	699	694.89	8.56×10^{-1}
11	853	864.36	7.05×10^{-1}	644	673.05	2.62×10^{-1}
12	230	831.28	$< \mathbf{10^{-4}}$	710	649.16	1.43×10^{-2}
13	523	641.55	$< \mathbf{10^{-4}}$	463	499.90	1.01×10^{-1}
14	919	570.53	$< \mathbf{10^{-4}}$	408	442.73	9.31×10^{-2}
15	149	498.78	$< \mathbf{10^{-4}}$	414	388.87	1.92×10^{-1}
16	706	543.25	$< \mathbf{10^{-4}}$	400	424.70	2.37×10^{-1}
17	284	469.06	$< \mathbf{10^{-4}}$	397	366.24	1.08×10^{-1}
18	309	497.54	$< \mathbf{10^{-4}}$	367	388.18	2.91×10^{-1}
19	1,520	414.62	$< \mathbf{10^{-4}}$	353	319.24	5.77×10^{-2}
20	576	384.47	$< \mathbf{10^{-4}}$	272	299.39	1.08×10^{-1}
21	370	247.31	$< \mathbf{10^{-4}}$	191	192.24	9.48×10^{-1}
22	249	247.7	8.96×10^{-1}	149	193.60	$\mathbf{1.40 \times 10^{-3}}$
All			$< \mathbf{10^{-4}}$			$< \mathbf{10^{-4}}$

Significant p-values are bolded (see *Methods*).

Table S2: Observed and expected outlier windows in intergenic regions for MD-M, IF-M, MD-F, and IF-F.

Method	Observed	Expected	Permutation p-value
MD-M	5,441	4,578.11	$< \mathbf{10^{-4}}$
IF-M	6,958	6,378.42	$< \mathbf{10^{-4}}$
MD-F	10,847	9,590.16	$< \mathbf{10^{-4}}$
IF-F	7,776	7,351.04	$< \mathbf{10^{-4}}$

Significant p-values are bolded (see *Methods*).

Table S3: Observed and expected outlier windows across four regions of protein-coding genes for MD-M, IF-M, MD-F, and IF-F.

Region	Observed	Expected	Permutation p-value	Observed	Expected	Permutation p-value
		Method: MD-M			Method: IF-M	
Exon	52	41.70	1.09×10^{-1}	60	69.68	2.55×10^{-1}
Intron	2,317	2,320.22	7.17×10^{-1}	3,912	3,875.89	$\mathbf{6.20 \times 10^{-3}}$
5′UTR	19	15.06	2.57×10^{-1}	20	25.25	3.44×10^{-1}
3′UTR	41	52.02	1.32×10^{-1}	63	84.18	1.66×10^{-2}
All			1.51×10^{-1}			$\mathbf{4.90 \times 10^{-2}}$
		Method: MD-F			Method: IF-F	
Exon	93	96.75	7.44×10^{-1}	69	83.82	1.02×10^{-1}
Intron	5,423	5,378.68	$\mathbf{5.70 \times 10^{-3}}$	4,697	4,657.45	$\mathbf{6.40 \times 10^{-3}}$
5′UTR	22	34.96	2.47×10^{-2}	19	30.33	3.24×10^{-2}
3′UTR	93	120.61	$\mathbf{8.80 \times 10^{-3}}$	91	104.4	1.85×10^{-1}
All			$\mathbf{9.40 \times 10^{-3}}$			$\mathbf{3.18 \times 10^{-2}}$

Significant p-values are bolded (see *Methods*).

Table S4: Observed and expected outlier windows in genomic regions with low GC content ($< 50\%$) for MD-M, IF-M, MD-F, and IF-F.

Method	Observed	Expected	Permutation p-value
MD-M	4,818	4,643.96	$\mathbf{1.00 \times 10^{-4}}$
IF-M	6,870	6,604.49	$< \mathbf{10^{-4}}$
MD-F	10,100	9,784.22	$< \mathbf{10^{-4}}$
IF-F	7,803	2,528.49	$< \mathbf{10^{-4}}$

Significant p-values are bolded (see *Methods*).

Table S5: Observed and expected outlier windows in repetitive regions for MD-M, IF-M, MD-F, and IF-F.

Method	Observed	Expected	Permutation p-value
MD-M	4,704	4,451.41	$< \mathbf{10^{-4}}$
IF-M	6,462	6,325.47	$\mathbf{1.22 \times 10^{-2}}$
MD-F	9,601	9,379.68	$\mathbf{8.00 \times 10^{-4}}$
IF-F	7,607	7,291.74	$< \mathbf{10^{-4}}$

Significant p-values are bolded (see *Methods*).

Table S6: Observed and expected outlier windows in genomic regions with low CRG scores (≤ 0.9) for MD-M, IF-M, MD-F, and IF-F.

Method	Observed	Expected	Permutation p-value
MD-M	1,101	495.34	$< \mathbf{10^{-4}}$
IF-M	809	697.33	$< \mathbf{10^{-4}}$
MD-F	1,390	1,051.58	$< \mathbf{10^{-4}}$
IF-F	868	817.11	1.47×10^{-1}

Significant p-values are bolded (see *Methods*).

Table S7: Observed and expected outlier windows across autosomes after removing repetitive regions and regions with low CRG scores (≤ 0.9) for MD-M, IF-M, MD-F, and IF-F.

Chr	Observed	Expected	Permutation p-value	Observed	Expected	Permutation p-value
	Method: MD-M			Method: IF-M		
1	278	243.74	2.24×10^{-2}	334	390.76	$\mathbf{1.50 \times 10^{-3}}$
2	72	279.73	$< \mathbf{10^{-4}}$	458	451	7.40×10^{-1}
3	131	226.96	$< \mathbf{10^{-4}}$	392	364.52	1.26×10^{-1}
4	240	229.38	4.45×10^{-1}	474	365.24	$< \mathbf{10^{-4}}$
5	256	201.91	$\mathbf{3.00 \times 10^{-4}}$	309	324.55	3.70×10^{-1}
6	328	218.84	$< \mathbf{10^{-4}}$	385	352.66	7.28×10^{-2}
7	116	188.82	$< \mathbf{10^{-4}}$	348	302.37	6.30×10^{-3}
8	414	182.45	$< \mathbf{10^{-4}}$	279	293.81	3.89×10^{-1}
9	169	139.72	1.27×10^{-2}	170	226.44	$\mathbf{1.00 \times 10^{-4}}$
10	123	169.95	$< \mathbf{10^{-4}}$	293	274.17	2.29×10^{-1}
11	180	155.25	3.74×10^{-2}	214	250.87	1.34×10^{-2}
12	14	146.48	$< \mathbf{10^{-4}}$	216	236.53	1.88×10^{-1}
13	201	124.86	$< \mathbf{10^{-4}}$	210	201.98	5.48×10^{-1}
14	182	105.61	$< \mathbf{10^{-4}}$	163	169.80	6.18×10^{-1}
15	32	92.42	$< \mathbf{10^{-4}}$	128	149.38	7.45×10^{-2}
16	39	101.74	$< \mathbf{10^{-4}}$	156	165.31	4.75×10^{-1}
17	52	86.59	$< \mathbf{10^{-4}}$	134	139.81	6.32×10^{-1}
18	116	99.23	9.14×10^{-2}	166	159.37	5.72×10^{-1}
19	253	60.58	$< \mathbf{10^{-4}}$	93	95.84	8.00×10^{-1}
20	6	72.45	$< \mathbf{10^{-4}}$	99	116.87	9.33×10^{-2}
21	1	47.89	$< \mathbf{10^{-4}}$	86	76.29	2.53×10^{-1}
22	18	46.39	$< \mathbf{10^{-4}}$	76	75.41	8.59×10^{-1}
All			$< \mathbf{10^{-4}}$			$< \mathbf{10^{-4}}$
	Method: MD-F			Method: IF-F		
1	577	571.02	7.72×10^{-1}	409	443.79	7.94×10^{-2}
2	239	655.05	$< \mathbf{10^{-4}}$	502	509.08	7.47×10^{-1}
3	289	531.76	$< \mathbf{10^{-4}}$	419	413.09	7.35×10^{-1}
4	618	537.25	$\mathbf{1.00 \times 10^{-4}}$	444	417.40	1.65×10^{-1}
5	739	472.88	$< \mathbf{10^{-4}}$	420	366.59	3.80×10^{-3}
6	515	513.78	9.24×10^{-1}	378	399.37	2.66×10^{-1}
7	583	442.18	$< \mathbf{10^{-4}}$	357	342.95	4.43×10^{-1}
8	566	427.36	$< \mathbf{10^{-4}}$	326	333.05	7.17×10^{-1}
9	347	327.29	2.49×10^{-1}	239	254.58	3.25×10^{-1}
10	397	398.08	9.62×10^{-1}	311	308.66	8.88×10^{-1}
11	402	363.61	3.91×10^{-2}	248	282.75	3.49×10^{-2}
12	74	343.01	$< \mathbf{10^{-4}}$	296	267.36	7.90×10^{-2}
13	194	292.39	$< \mathbf{10^{-4}}$	197	227.68	3.71×10^{-2}
14	352	247.46	$< \mathbf{10^{-4}}$	193	191.93	8.89×10^{-1}
15	70	216.69	$< \mathbf{10^{-4}}$	185	168.87	2.05×10^{-1}
16	359	238.23	$< \mathbf{10^{-4}}$	171	186.05	2.82×10^{-1}
17	72	202.78	$< \mathbf{10^{-4}}$	174	158.21	1.99×10^{-1}
18	176	232.37	$\mathbf{1.00 \times 10^{-4}}$	194	180.95	3.02×10^{-1}
19	471	141.95	$< \mathbf{10^{-4}}$	112	109.50	7.68×10^{-1}
20	242	169.66	$< \mathbf{10^{-4}}$	129	131.77	8.24×10^{-1}
21	175	112.52	$< \mathbf{10^{-4}}$	85	87.41	8.38×10^{-1}
22	89	108.67	5.58×10^{-2}	77	84.95	3.81×10^{-1}
All			$< \mathbf{10^{-4}}$			$\mathbf{4.75 \times 10^{-2}}$

Significant p-values are bolded (see *Methods*).

Table S8: Observed and expected outlier windows in intergenic regions after removing repetitive regions and regions with low CRG scores (≤ 0.9) for MD-M, IF-M, MD-F, and IF-F.

Method	Observed	Expected	Permutation p-value
MD-M	1,829	1,617.05	$< \mathbf{10^{-4}}$
IF-M	2,745	2,502.52	$< \mathbf{10^{-4}}$
MD-F	4,256	3,732.47	$< \mathbf{10^{-4}}$
IF-F	3,009	2,837.44	$< \mathbf{10^{-4}}$

Significant p-values are bolded (see *Methods*).

Table S9: Observed and expected outlier windows across four regions of protein-coding genes after removing repetitive regions and regions with low CRG scores (≤ 0.9) for MD-M, IF-M, MD-F, and IF-F.

Region	Observed	Expected	Permutation p-value	Observed	Expected	Permutation p-value
	Method: MD-M			Method: IF-M		
Exon	40	38.89	7.61×10^{-1}	50	63.46	9.03×10^{-2}
Intron	1,203	1,179.05	$\mathbf{8.70 \times 10^{-3}}$	1,964	1,924.67	$\mathbf{3.00 \times 10^{-4}}$
5′UTR	14	12.91	5.95×10^{-1}	14	21.14	1.17×10^{-1}
3′UTR	15	41.15	$< \mathbf{10^{-4}}$	46	64.73	1.35×10^{-2}
All			$< \mathbf{10^{-4}}$			$\mathbf{1.06 \times 10^{-2}}$
	Method: MD-F			Method: IF-F		
Exon	85	88.66	7.29×10^{-1}	64	74.65	2.37×10^{-1}
Intron	2,735	2,688.05	$\mathbf{1.10 \times 10^{-3}}$	2,299	2,262.64	$\mathbf{6.70 \times 10^{-3}}$
5′UTR	20	29.46	8.22×10^{-2}	15	24.83	3.89×10^{-2}
3′UTR	60	93.82	2.00×10^{-4}	63	78.87	7.40×10^{-2}
All			$\mathbf{8.00 \times 10^{-4}}$			$\mathbf{2.65 \times 10^{-2}}$

Significant p-values are bolded (see *Methods*).

Table S10: Observed and expected outlier windows with low GC content ($< 50\%$) after removing repetitive regions and regions with low CRG scores (≤ 0.9) for MD-M, IF-M, MD-F, and IF-F.

Method	Observed	Expected	Permutation p-value
MD-M	1,914	1,875.54	1.73×10^{-1}
IF-M	3,173	3,012.68	$\mathbf{1.00 \times 10^{-4}}$
MD-F	4,545	4,394.03	$\mathbf{3.00 \times 10^{-4}}$
IF-F	3,523	3,412.28	$\mathbf{3.10 \times 10^{-3}}$

Significant p-values are bolded (see *Methods*).
